# Not only COVID-19 disease impacts ambulance emergency demands but also lockdowns and quarantines

**DOI:** 10.1186/s12873-023-00772-3

**Published:** 2023-01-13

**Authors:** Séverine Vuilleumier, Thierry Spichiger, Sandrine Dénéréaz, Assunta Fiorentino

**Affiliations:** 1 La Source School of Nursing, University of Applied Sciences and Art Western Switzerland (HES-SO), Lausanne, CH-1004 Switzerland; 2 ES ASUR, Vocational Training College for Registered Paramedics and Emergency Care, Le Mont- sur-Lausanne, CH-1052 Switzerland

**Keywords:** Ambulances, COVID-19, Emergency paramedic, Emergency medical services, Emergency medicine, Health, Pandemics

## Abstract

**Background:**

The pandemic has impacted both patients infected by the SARS-CoV-2 virus and patients who seek emergency assistance due to other health issues. Changes in emergency demands are expected to have occurred during the pandemic, the objective of this investigation is to characterize the changes in ambulance emergency demands during the first year of the COVID-19 pandemic in the Vaud State of Switzerland. The goal of this research is to identify the collateral effects of the COVID-19 pandemic on emergency demands. To do so, this study quantifies the differences in health issues, level of severity, and patients’ sociodemographic characteristics (age, location, gender) prior to and during the outbreak.

**Method:**

This is a retrospective, descriptive and comparative statistical analysis of all ambulance emergency missions from 2018 to 2020 (*n* = 107,150) in the State of Vaud in Switzerland. Variables analyzed were the number of ambulance missions, patient age and gender, health issues, severity (NACA scores), number of non-transports, mission times and locations. Variables were compared between prepandemic and pandemic situations across years and months. Comparative analysis used bivariate analysis, χ2 test, Student’s t test, and Mann‒Whitney U test.

**Results:**

The pandemic has had two major impacts on the population’s emergency demands. The first appears to be due to COVID-19, with an increase in respiratory distress cases that doubled in November 2020. The second relates to the implementation of lockdown and quarantine measures for the population and the closures of restaurants and bars. These might explain the decrease in both the number of traumas and intoxications, reaching more than 25% and 28%, respectively. An increase in prehospital emergency demands by the older population, which accounted for 53% of all demands in 2020, is measured.

**Conclusion:**

Collateral effects occurred during 2020 and were not only due to the pandemic but also due to protective measures deployed relative to the population. This work suggests that more targeted reflections and interventions concerning the most vulnerable group, the population of people 65 and older, should be of high priority. Gaining generalizable knowledge from the COVID-19 pandemic in prehospital settings is critical for the management of future pandemics or other unexpected disasters.

## Background

The virus causing severe acute respiratory syndrome SARS-CoV-2 (COVID-19), at the origin of a worldwide pandemic, might have several consequences on patients with other emergency needs [1–3]. For example, in Germany, a study found a massive drop of 63.8% in pediatric emergency health care utilization [4]. A drastic reduction in the number of patients seeking medical help has also been documented for cardiovascular diseases [5] and stroke (78% versus 57%, *p* < 0.001) in southern Europe [1]. Previous coronavirus outbreaks resulted in drastic changes in utilization of the health care system [6, 7]. Other health or social issues might be a concern and still need to be unveiled.

One challenge for a health system is maintaining accessibility to quality care for the entire population [8]. Emergency medical services (EMS) hold a predominant position in maintaining the population’s access to health care. In Switzerland, over 20% of admissions in an emergency department occur via ambulance [9, 10]. EMS provide continuous (24 h a day) and diversified care (diseases and traumas) to the entire population [8]. In 2015, 460,000 assistance and rescue missions occurred throughout Switzerland [11].

In a recent study, Vuilleumier et al. [12] evaluated emergency medical services in the state of Vaud in Switzerland. This study showed that life-threatening emergencies concern only 13% of missions; the remaining, i.e., 87%, concern non-urgent missions. The latter typically involve elderly persons with complex pathologies (multiple diseases or chronic diseases) or non-specific problems such as decreased general health conditions (7.7%) or mental health concerns (6.9%). Vuilleumier et al. [12] also show that over half of the patients requesting emergency services were 65 or older. Nearly 12% of EMS responses resulted in the non-transport of the patient, and only 23% concerned trauma.

COVID-19 has generated, and is still generating, a stressful situation for the population and for all health care systems. In this research, it is hypothesized that the COVID-19 pandemic is disruptive for prehospital health care, and collateral damage is expected for non-COVID-19 patients’ needs. This effect might have been amplified during periods with lockdown and social distance measures. A better description of the population using emergency medical services during the pandemic in terms of patients’ age categories and health issues is important as well as a quantification of changes compared to years prior to the COVID-19 pandemic, i.e., COVID-19-free years. This knowledge is critical not only for the patient and the emergency departments but also for the entire organization of prehospital activities. Such analysis is also of interest to hospitals that have direct repercussions of ambulance transport through admissions to the emergency department, which might impact patient safety as well as patient flow [13–16].

During the first wave of the pandemic in the state of Vaud [17], the Swiss government (Federal Council) implemented an emergency response and deployed some social distancing measures for the population. With the first positive case on March 2, 2020, they were issued in March 2020 until June 2020 and consecutively in October 2020. These measures include lockdown (semi confinement), quarantines, social distancing, travel restrictions and nonurgent planned care procedures were postponed. These protective measures for the population are also expected to impact ambulance emergency demands.

Here, it is assumed that the COVID-19 pandemic is disruptive for prehospital health care, and changes are also expected for non-COVID-19 patients’ needs. In this context, this study aims to uncover significant changes in the number of patients seeking medical help given a specific health issue and their characteristics, with the objective of detecting collateral effects or damages. Following Montaner et al., Sher et al. and Sung et al., it is referred here to COVID-19 collateral damage as the “unwelcome consequences of COVID-19 for individuals, families, communities, and society” [1–3]. They will be the object of careful interpretation, as only differences in number will be provided that are not directly linked to causes. The specific objectives are (i) to characterize the population requesting emergency health services via ambulance services (including age, gender, health issue type and severity, time and location) and (ii) to identify significant changes in these variables regarding the COVID-19 pandemic. Based on the results, the aim is to gain knowledge regarding changes in population emergency demands during an epidemic. The results of this study will provide key information to develop new recommendations for preparedness in prehospital settings in Switzerland.

## Methods

### Context

This study was conducted in the State of Vaud in Switzerland (approximately 800,000 inhabitants), where eight ambulance services (both private and public) are appointed by one emergency dispatch center (144) 24 h a day for rescue and assistance missions. Priority level is assigned by the centralized prehospital medical telephone dispatch center. Since 2018, every on-site intervention has been documented by the ambulance crew, and the collected data concern the patient (e.g., age, gender, type and severity of health issue) and the intervention (e.g., time or place). All this routine information is entered and stored in a database called “Attrib”, which is owned and managed by the Directorate-General for Health of the State of Vaud (DGS). This project has been done in close collaboration with the DGS, which provides complete access to relevant data and offers full support to this research project.

### Data

The data on ambulance missions cover the period from 1 to 2018 to 31 December 2020. Only primary missions are considered for the analysis; these concern the care of patients whose lives are in immediate danger from the place of intervention to arrival at a suitable health institution. The recorded 107,150 ambulance missions were anonymized. The variables analyzed for all missions are the age and gender of the patient, the NACA (National Advisory Committee for Aeronautics) score, the main health issue (problem code), the time of mission, and nontransports (see the detailed description below).

The NACA score indicates the degree of severity of traumas and illnesses; it is widely used at the international level and in Switzerland by prehospital health care providers [18–21]. The NACA score is a scale with 9 values (0–7; 9), the minimum score being 0 (unscathed patient) and the maximum 7 (deceased patient); a value of 9 indicates cancelled missions without contact with the patient. A NACA score of 3 is the threshold indicating that a patient requires hospital investigation and treatment with the trauma or illness being nonlife threatening. A NACA score of 4 means that without hospital treatment, the disease or lesions could evolve toward vital risk, for example, a myocardial infarction. NACA scores between 4 and 6 can be considered “urgent” cases, and cases with NACA scores between 0 and 3 can be considered “nonurgent” [12]. The health issue is described according to 33 predefined categories identified by the paramedics: for example, trauma, cardiac arrest or intoxication (Table 1). Finally, nontransport determines whether the patient was taken to the hospital by the paramedics. Emergency calls that did not result in an ambulance intervention were not considered in the analysis.

### Statistics

This project performed a deep descriptive and statistical analysis of the database on ambulance missions. The following variables are evaluated: number of primary missions, age, sex ratio, NACA score (number and percentage), typology of health issues (33 categories), location of mission, number of missions per hour, and nontransported patients (number, percentage, age, NACA score and causes). Values are analyzed over the years, months and weeks depending on the time frame of the analyses performed. Continuous variables are presented as numbers and frequencies (%). Comparative analysis uses bivariate analysis, as appropriate the χ2 test. A *p* < 5% statistically significant difference was considered. All statistical analyses were performed using R.

The investigation concerned the number of primary missions, the number and percentage of NACA scores, the proportion of men and women, the proportion of health issues, the proportion of nontransported patients and the distribution of times of missions over 24 h. For all the above-mentioned investigations, their values for each year and the differences observed across years are evaluated. The age of the patients was considered across 3 categories of age: 0 and 16 years old, 17 and 64 years old, and 65 years old and more. Those age categories are selected to distinguish the pediatric population, adult population and senior population and are not of equal size. For the 33 documented health problems, the difference in the number of cases month by month considering the average values found in 2018 and 19 compared to the value in 2020 is considered.

## Results

### Number of primary interventions in 2018, 2019 and 2020, patient sex, age and severity

An increase in the total number of primary missions close to 2% is observed in 2019 compared to 2018, while a much lower increase is observed between 2019 and 2020 (Table 1). However, between these years, the proportion of ambulance missions across group ages significantly changes. The percentage of primary missions concerned by patients aged 65 years and older increased significantly. The percentage increased from 50.64 to 53% in 2020 (using a 3-sample test for equality of proportions without continuity correction, data show X2 = 33.871, df = 2, *p < 0.001*). At the same time, there is a significant decrease in missions concerning the 0–16 age group between 2019 and 2020, which is reduced from 6.3 to 5.18% in 2020 (X2 = 36.05, df = 2, *p < 0.001*). A decrease in the age Group 17–64 is also observed but only between 2019 and 2020.

**Table 1:** Health issues with the number and percentage of cases for 2018, 2019 and 2020 in the state of Vaud, Switzerland

Health Issues	2018		2019		2020	
	**Number**	**%**	**Number**	***%***	**Number**	***%***
**Cardiac arrest (CA)**	625	*1,78*	579	*1,61*	612	*1,70*
**Nontraumatic coma**	167	*0,47*	138	*0,38*	151	*0,42*
**Alertness disorders**	766	*2,18*	717	*2,00*	629	*1,74*
**Brief loss of consciousness, noncardiac malaise (vagal, hypoTA)**	1977	*5,62*	1995	*5,56*	1964	*5,44*
**Convulsive seizure**	917	*2,61*	921	*2,57*	891	*2,47*
**Respiratory distress or failure**	2439	*6,93*	2536	*7,07*	2832	*7,85*
**Asthma attack**	36	*0,10*	33	*0,09*	33	*0,09*
**Heart attack, nontraumatic chest pain (conscious victim)**	1628	*4,63*	1636	*4,56*	1741	*4,82*
**Shock (hypovolemic cardiogenic, septic, anaphylactic)**	264	*0,75*	291	*0,81*	306	*0,85*
**Hemorrhage without trauma (digestive, ENT, gynecological)**	582	*1,65*	682	*1,90*	641	*1,78*
**Rhythm and/or conduction disorders (bradycardia, AV block, …)**	460	*1,31*	437	*1,22*	455	*1,26*
**Hypertensive emergency**	306	*0,87*	271	*0,76*	282	*0,78*
**Neurological deficit without coma and nontraumatic**	911	*2,59*	973	*2,71*	1010	*2,80*
**Headaches**	336	*0,95*	397	*1,11*	320	*0,89*
**Psychiatric cases (agitation, anxiety, …)**	2423	*6,89*	2326	*6,48*	2493	*6,91*
**Intoxication without coma (OH, drugs, CO, smoke)**	2131	*6,06*	2151	*5,99*	1994	*5,53*
**Allergy (without anaphylactic shock)**	248	*0,70*	190	*0,53*	255	*0,71*
Polytrauma*	130	*0,37*	131	*0,37*	127	*0,35*
Trauma to limbs (including dislocation)*	4347	*12,36*	4467	*12,45*	4425	*12,26*
Craniocerebral trauma*	1616	*4,59*	1753	*4,89*	1701	*4,71*
Maxillofacial trauma*	647	*1,84*	699	*1,95*	576	*1,60*
Trauma to the spine*	648	*1,84*	610	*1,70*	500	*1,39*
Thoracic trauma*	424	*1,21*	425	*1,18*	372	*1,03*
Abdominal trauma*	88	*0,25*	78	*0,22*	83	*0,23*
**Trauma to the pelvis (perineum)** *	108	*0,31*	139	*0,39*	116	*0,32*
**Nontraumatic abdominal pain**	1987	*5,65*	2014	*5,61*	1987	*5,51*
**Nontraumatic lumbar pain**	658	*1,87*	677	*1,89*	624	*1,73*
**Health care impossible at home**	612	*1,74*	792	*2,21*	964	*2,67*
**Declined general condition (BEG)**	2702	*7,68*	2982	*8,31*	3409	*9,45*
**Pregnancy, childbirth, birth**	135	*0,38*	114	*0,32*	117	*0,32*
**Burns**	61	*0,17*	45	*0,13*	48	*0,13*
**Drowning without CA**	6	*0,02*	5	*0,01*	3	*0,01*
**Electrification without CA**	8	*0,02*	6	*0,02*	10	*0,03*
**Hypothermia without CA**	28	*0,08*	23	*0,06*	20	*0,06*
**Other**	4763	*13,54*	4649	*12,96*	4393	*12,17*
**Total**	35,184		35,882 *(+ 1,98%)*		36,084 *(+ 0,56%)*	

^*^Indicates the health issues aggregated in one trauma category for further analysis. Note that the proportion of women was higher when evaluating the sex ratio in 2018 and 2019 (proportion test with *P* ≤ 0.01 and *P* ≤ 0.001, respectively)

When looking at the degree of severity measured by the NACA score, it is found in 2020 that the number of urgent primary missions, i.e., with NACA scores ranging from 4 to 7 indicating a life-threatening emergency, increases significantly during the pandemic period (Table 2). At the same time, the proportion of nonurgent primary missions, i.e., with NACA scores ranging from 0 to 3 indicating a nonlife-threatening emergency, decreases in 2020 but still represents 86,7% of the primary missions. When considering the different age groups and the degree of severity, the percentages of the age Group 65 years and older increase regardless of the category related to the degree of severity. Conversely, the proportions of the other two age groups decrease between 2018 and 2020, with a significant decrease in the 0–16 age group, primarily during the pandemic year 2020.

**Table 2:** Number of ambulance missions in 2018, 2019, 2020 according to different age groups and severity scores. Some missions were undocumented regarding age, leading to missing values (NA = 28, 38, 20 in resp. 2018, 2019, 2020)

Age groups	**2018**			**2019**			**2020**		
	**Total**	**Nonurgent**	**Urgent**	**Total**	**Nonurgent**	**Urgent**	**Total**	**Nonurgent**	Urgent
**0–16**	***2160 (6,14%)***	*1968 (6,4%)*	*192 (4,2%)*	***2153 (6,01%)***	*1978 (6,3%)*	*175 (3,9%)*	***1867 (5,18%)***	*1708 (5,5%)*	*159 (3,3%)*
**17–64**	***15,193 (43,22)***	*13,454 (43,9%)*	*1739 (38,4%)*	***15,264 (42,58%)***	*13,604 (43,9%)*	*1660 (36.8%)*	***15,082 (41,82%)***	*13,360 (42,7%)*	*1722 (35,8%)*
**65 +**	***17,803 (50,64%)***	*15,211 (49,7%)*	*2592 (57,3%)*	***18,428 (51,41%)***	*15,753 (50,3%)*	*2675 (59,3%)*	***19,115 (53,00%)***	*16,186 (51,8%)*	*2929 (60,9%)*
**Total**	***35,156***	*30,633 (87,1%)*	*4523 (12,9%)*	***35,845***	*31,335 (87,4%)*	*4510 (12,6%)*	***36,064***	*31,254 (86,7%)*	*4810 (13,3%)*

Patients aged 65 years and older were more frequently found in the urgent (NACA 4–7) category than in the nonurgent category (NACA 0–3) (all tests were significant with *p < 0.001* with X^*2*^ = 92.002, 128.61, 138.38 for 2018, 2019 and 2020, respectively, using Pearson’s chi-squared test with Yates’ continuity correction).

Proportions of men and women were equal in 2020 but differed slightly in 2018 and 2019, with an excess of women (X2 = 8.2628, df = 1, *p < 0.01* and X2 = 29.589, df = 1, *p < 0.001*, respectively, using the 1-sample proportions test with continuity correction). The proportion of nontransported patients increased from 11.7 to 12.3% between 2018 and 2020 (Table 3).

**Table 3:** Number of missions that resulted in nontransport of the patient

	Number of nontransport (percentage)	Total number of missions
2018	4135 (11,76%)	35,156
2019	4318 (12,05%)	35,845
2020	4447 (12,33%)	36,064

**Fig. 1:**
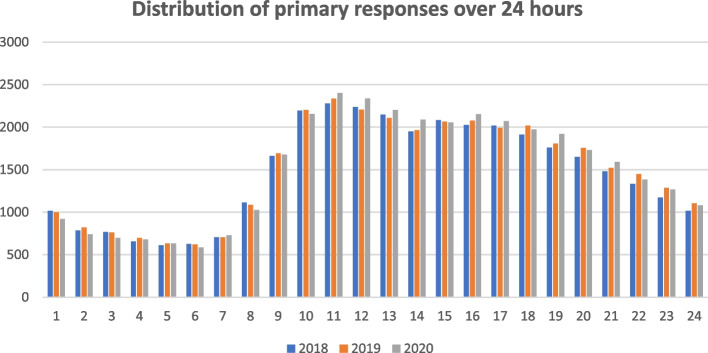
Distribution of primary responses over 24 h for 2018, 2019 and 2020

The distributions of primary missions over 24 h follow a similar pattern for all years (Fig. 1), with no exception during the pandemic: a growing phase starting at 7 am to a maximum of interventions between 11:00 a.m. and 4 p.m. Then, a continual decrease occurs until 7 am.

### Health problems: comparison between the average for 2018–2019 compared to 2020

For documented health problems (Table 1), the difference in the number of cases month by month was evaluated. For each month, the difference is computed based on the average values of 2018-19 against 2020. Differences are expressed as percentages of increase and decrease. Only health issues with significant changes are displayed in Table 4 with the percentage of increase or decrease as well as p- values presented.

**Table 4:** Changes in the number of cases per health issue when comparing months of COVID-19-free years (average between 2018–2019) vs. 2020. The significance of changes in the number of cases per health issue when comparing months of COVID-19-free years (average between 2018–2019) vs. 2020 is evaluated using X2

Health issue	January		February		March		April		May		June		July		August		September		October		November		December	
	*%*	*p*	*%*	*p*	*%*	*p*	*%*	*p*	*%*	*p*	*%*	*p*	*%*	*p*	*%*	*p*	*%*	*p*	*%*	*p*	*%*	*p*	*%*	*p*
**Brief loss of consciousness, non-cardiac malaise (vagal, hypoTA)**	26,8	*< 0,05*	-12,0	-	-3,2	*-*	-22,3	*-*	-24,8	-	-3,6	-	0,3	-	2,7	*-*	6,6	-	38,1	*-*	-3,6	*-*	-8,8	*-*
**Convulsive seizure**	-0,6	*-*	34,7	-	2,3	*-*	-19,0	*-*	0,0	-	-22,6	-	3,4	-	-28,0	*< 0,05*	5,5	-	-4,7	*-*	2,3	*-*	-6,3	*-*
**Respiratory distress or failure**	-26,6	*< 0,001*	-8,0	-	45,6	*< 0,001*	-2,5	*-*	-23,6	-	-5,9	-	-7,5	-	9,8	*-*	21,7	-	36,2	*-*	100,5	*< 0,001*	27,1	*< 0,05*
**Heart attack, non-traumatic chest pain (conscious victim)**	-13,9	*-*	0,8	-	-1,3	*-*	31,0	*< 0,01*	-7,0	-	-1,1	-	7,4	-	9,2	*-*	20,2	-	9,6	*-*	15,0	*-*	14,0	*-*
**Headaches**	-10,1	*-*	-25,4	-	-30,0	*-*	-10,6	*-*	-5,1	-	-15,2	-	-43,3	-	-38,5	*-*	49,1	-	-37,1	*< 0,05*	13,8	*-*	14,3	*-*
**Intoxication without coma (OH, drugs, CO, smoke)**	15,3	*-*	2,3	-	-24,6	*< 0,05*	-19,4	*-*	7,7	-	1,3	-	-1,6	-	2,8	*-*	-1,6	-	-9,4	*< 0,05*	-25,8	*< 0,001*	-28,7	*< 0,001*
**Allergy (without anaphylactic shock)**	8,3	*-*	36,0	-	-3,4	*-*	-17,6	*-*	18,9	-	-27,3	-	25,5	-	69,8	*< 0,05*	14,9	-	-6,3	*-*	2,7	*-*	44,8	*-*
**Trauma**	6,0	*-*	9,5	-	-20,0	*< 0,001*	-25,019128	*< 0,001*	-9,3	-	-10,526316	-	6,6	-	2,67	*-*	1,8	-	15,6	*-*	-12,3	*< 0,001*	-4,6	*-*
**Unable to care at home**	29,0	*-*	30,5	-	41,1	*-*	29,2	*< 0,05*	-7,8	-	31,2	-	33,9	-	30,7	*-*	30,8	-	96,3	*< 0,01*	38,6	*-*	72,2	*< 0,01*
**Decreased general condition**	2,0	*-*	8,1	-	27,0	*< 0,01*	15,3	*< 0,01*	-4,3	-	13,2	-	12,6	-	12,1	*-*	12,1	-	52,2	*< 0,01*	75,1	*< 0,001*	15,5	*-*

**Fig. 2:**
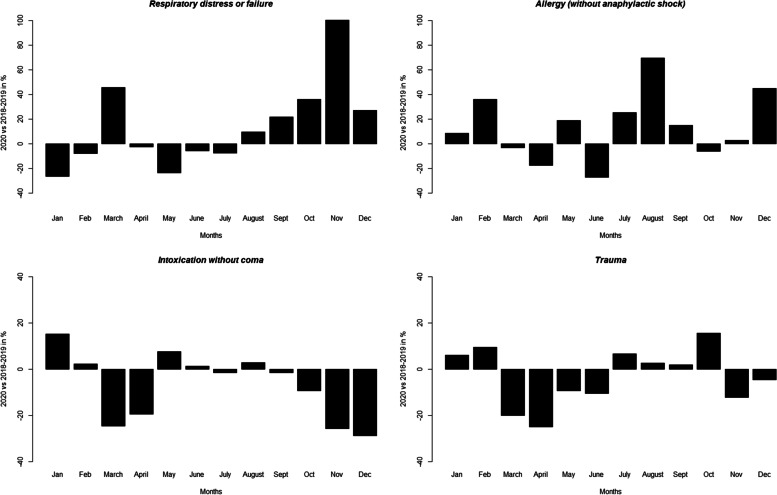
Difference in the percentage of cases between 2018–2019 (COVID-19-free years) and 2020. Panel **a** concerns respiratory distress or failure, **b** allergy (without anaphylactic shock), **c** intoxication without coma, and **d** trauma. Differences were evaluated using the X2 test and are highlighted as follows: *p* ≤ 0.05, ** *p* ≤ 0.01, *** *p* ≤ 0.001

The results of this analysis show two main trends of a significant increase or decrease in health issues. Increases in certain health problems during the COVID-19 pandemic have been observed, including respiratory distress or failure, declined general condition, healthcare impossible at home, and allergies. The number of “respiratory distress or failure” cases increased significantly during March, November, and December 2020, following the peak of epidemic waves (Fig. 2). The increase is particularly high during the second epidemic wave, with more than 100% increases in cases. This value reaches 40% during the first wave. Additionally, a significant increase (more than 60%) in allergies leading toward ambulance intervention was observed in August. The declined general condition follows a similar trend with a significant increase of 27% in March that reaches 52% and 75% in October and November, respectively. Health care impossible at home was particularly high during the second wave, with an increase of 96% and 72% of the cases in October and December, respectively. Heart attack as well as brief loss of consciousness also show a significant increase at the beginning of the year. Interestingly, significant decreases in cases of multiple health problems were also observed (Fig. 2). Among them, two categories changed at a very high level: trauma and intoxication without coma (alcohol, drugs, smoke, carbon monoxide). Both decreased significantly during the epidemic waves, with approximately 25% fewer intoxications in March, April, November and December, and traumatic injuries were reduced by 12–25% in the same periods.

## Discussion

The COVID-19 pandemic has major repercussions on various health domains, such as a significant increase in population mortality and a decrease in life expectancy in most European countries [22, 23]. Looking at the changes and variations in the prehospital emergency demands before and during the COVID-19 pandemic, changes in the patient population requesting an ambulance during 2020 are found and are significant. This is consistent with what was expected, as ambulance emergency demands due to COVID-19 disease symptoms increased. Interestingly, collateral effects that are not related to the disease itself are also found and appear to relate to government protective measures. Changes vary according to age groups, health issues and severity of a health problem.

### Both pandemic and social distancing measures impact EMS demands

Several health issues increased during the COVID-19 pandemic. A concordance between health issues related to respiratory distress and the successive epidemic waves in March and October 2020 is observed. Such concordance can be explained by predominantly respiratory symptomatology in COVID-19 and has been found in other studies (e.g. Satty et al. [24]). Additionally, from the start of the epidemic, an increase of at least 20% in ambulance transport due to the impossibility of treating patients in their home is observed: it peaks at a 96% increase in October 2020 and highlights the challenges of taking care of nonurgent but vulnerable individuals.

Interestingly, in 2020, there was a significant decrease of more than 20% in trauma and intoxication in March and April 2020 compared to the average value in 2018–2019. The decrease in the trauma category can be explained by the implementation of a semiconfinement that was associated with governmental restrictions such as semiconfinement, home office, school closure, bar and restaurant closures, and recommendations of restriction of many activities such as driving, traveling, or engaging in high risk sports (skiing or mountain biking, for instance). This trend has also been observed in various countries, such as Israel [25] and Finland [26, 27]. In Switzerland, a significant decrease in major trauma arriving at the emergency department has been measured during the governmental restriction phases [28]. The causes of trauma related to traffic accidents, for example, decreased by 17% compared to 2019 [28]. The measured reduction in cases of intoxication is certainly related to the complete or partial closure of bars, restaurants, and nightclubs as well as limitations of private gatherings during the epidemic’s waves in 2020. A similar trend was reported in Israel during the first lockdown period in March and April 2020, with a 23.65% decrease in overdoses compared with the previous year [25], as well as in Finland, with a decrease in alcohol-related intoxications [27].

The significant excess of cases of allergy in August might be related to both the environment and protective measures to the population. First, good meteorological conditions for outdoor activities occurred in Switzerland, and the second protective measure encouraged the population to spend time in the countryside and natural area, as all public places for entertainment were closed. However, such a relationship might require further analysis to be proven.

### Seniors where the most impacted

An increase in the number of primary missions from the population aged 65 years and older is measured. This increase is mainly observed in the following health problems: health care impossible at home and declined general condition. This could probably be explained by the reduced availability of family caregivers during the semicontainment period (working at home with dependent children, fear of transmitting the virus, gathering of people prohibited, etc.) and on the other hand by a decrease in social and health services offered to the older population, such as animations or meals in community and closure of senior day care centers. Fear of infection might have influenced the use of general health services and informal care [25]. It is also possible that existing vulnerabilities in the senior population were exacerbated during the pandemic. Such a trend has been observed in several studies that found a significant decrease in the senior population in the emergency department during epidemic waves [29, 30].

### Reduced part of transported patients

The number of nontransported patients increased in 2020. These findings are reported in many countries during the COVID-19 pandemic with varying proportions depending on the pandemic context and health authority responses. In Israel, the share of nontransported workers increased from 13.4% to 2019 to 19.9% in 2020. The authors suggest that this increase may be related to patients’ fear of possible SARS-CoV-2 related contamination if transported to the hospital during the height of the pandemic. In addition, the authors point out that these nontransports may have been the cause of a deterioration in the health status of patients who refused transport until the later stages of illness [25]. Another Finnish study also found an increase in nontransported patients during the first COVID-19 epidemic wave from 36.1% to 2019 to 39.9% in 2020 for the same period analyzed (*P* < 0.001) [31]. Another American study conducted in the state of Pennsylvania also found a 48% relative increase in nontransports during the first pandemic period [24].

### Targeted services might be the key

When looking at EMS demands and missions during the pandemic, the population aged 65 years and more appears to be the most affected by both the pandemic and social distancing measures. The significant increase in the health issues category “Declined general condition” and “Health care impossible at home” both might signal a leak of support of this population at their home. Indeed, caregivers (family members or friends) might not have been available for seniors due to many factors, such as school closures, quarantines, and fear of infecting senor individuals. Additionally, many care centers, which take care of approximately 2100 persons in the canton on a yearly basis, were closed during the pandemic, resulting in less professional surveillance during the daytime [32]. Additionally, general health check-ups and follow-up of chronic conditions were temporarily cancelled or replaced by telemedicine. Such results point out the importance of the community of caregivers, the small structures that offer meals and activities for the senior community. It might thus signal a need for targeted intervention for seniors during pandemics. The greater representation of this population also uncovers needs in terms of training for the ambulance crew that require skill to take care of complex situations and adapt interventions to their needs.

A recently published editorial stresses the importance of reflecting on the integration of emergency medical services in a more community-based approach centered on the principle of regionalization of resources and care networks in the interests of efficiency [33]. Among others, they highlight as a previous study [12] a need for a better match between ambulance emergency demands (social, health issues, severity, age) and ambulance emergency resources (workload, location, skills required). This might be a necessary step for an adaptive response in handling complex emergency demands during the pandemic.

Prevention measures and follow-up of chronic conditions could be considered and targeted to certain categories of the population during future pandemics. The prevention of risks related to vulnerability factors such as age should be considered relative to these research results, with, for example, the establishment of a support and follow-up network for the population aged 65 and over. Such actions could also have many additional benefits and prevent the currently observed unnecessary use of emergency departments by old patients [34–37]. A structured collaboration between paramedics, nurses, social emergency services, psychiatric emergency services, home care and palliative care might provide an adapted response to future pandemics and to other disasters related to climate change.

This research provides critical information on prehospital activity that does not benefit from large-scale monitoring, although detailed good-quality data are available to public authorities. Indeed, statistics on emergency demands by the population are generally missing in Switzerland. For example, the recent report on the impact of the COVID-19 pandemic on health care services in 2020 [38] did not mention prehospital-associated community services and their professionals, which include ambulance services, nurses, social emergencies, psychiatric emergencies, and palliative care. Little is known about the consequences of the pandemic on their activity and their patients. They all appear critical to an adapted and targeted prehospital health care system. Bridging the gap between researchers, clinicians, decision-makers, and available data is of critical importance to better prepare for future epidemic events or other natural disasters.

### Limitations of the research study

Generalization of our findings should be made with caution, as our study does have some limitations. First, implemented emergency responses to the pandemic and deployed social distancing measures for the population were specific to Switzerland. For example, gathering was prohibited and teleworking was generalized during lockdown (semiconfinement), but the population was allowed to do outdoor activities. Second, information bias might exist, as the collected data are concerned with emergency demands made via dispatch emergency centers where an ambulance was requested and might thus not represent all the emergency demands that occurred during the studied period. Third, the health issues considered have been identified during the emergency mission by the ambulance crew and are not a formal health diagnostic. Finally, although the amount of data available is very large, when comparing years (here only 3 years) and months across years, the sample size is markedly reduced. Consequently, detecting a true effect is reduced due to the low statistical power.

## Conclusion

Considering emergency demands for ambulances, collateral effects of the COVID-19 pandemic were observed during 2020. These collateral effects were not only due to the pandemic but also due to protective measures deployed for the population, such as lockdowns and quarantines. Interdiction of gathering and fears of being contaminated and contaminating relatives might also have played a role. Large and significant changes in demands are measured concerning trauma, intoxication and a considerable increase in the ability to stay home and a decrease in general condition. The most vulnerable group appear to be among those 65 and over. This population should be of high priority during pandemics or other unexpected disasters, and additional targeted interventions should be developed accordingly.

## Data Availability

The data that support the findings of this study are available from the Directorate-General for Health of the State of Vaud (Direction Générale de la Santé), but restrictions apply to the availability of these data, which were used under license for the current study and are not publicly available. Data as presented here in a yearly aggregated form are, however, available from the corresponding authors upon reasonable request and with permission of the Directorate-General for Health of the State of Vaud. All raw data belong to a database called “Attrib”, which provides an inventory of all the primary and secondary missions of the State of Vaud. Requests should be sent to the Directorate-General for Health of the State of Vaud.

## Abbreviations

NACA score: National Advisory Committee for Aeronautics score
EMS: Emergency Medical Services
COVID-19: Severe acute respiratory syndrome caused by SARS-CoV-2
